# Optimization of banana sucker production using different macropropagation structures and substrates

**DOI:** 10.1371/journal.pone.0352099

**Published:** 2026-06-22

**Authors:** Abel Sefasi, Mvuyeni Nyasulu, Khumbo Pute, Rowland Maganizo Kamanga, Eviness Nyalugwe, Lameck Matiki, Yamikani Gada, Joseph Chimungu

**Affiliations:** 1 Horticulture Department, Lilongwe University of Agriculture and Natural Resources, Bunda College, Lilongwe, Malawi; 2 Crop and Soil Sciences Department, Lilongwe University of Agriculture and Natural Resources, Bunda College, Lilongwe, Malawi; 3 Department of Crop Development, Ministry of Agriculture, Lilongwe, Malawi; Nuclear Science and Technology Research Institute, IRAN, ISLAMIC REPUBLIC OF

## Abstract

Banana (*Musa spp*.) production in Malawi is constrained by limited access to high-quality planting materials, restricting farm productivity. This study evaluated the effects of macro-propagation structures and substrates on growth and sucker production of banana cv. Williams, and examined the relationship between decapitated primary suckers and secondary sucker production. Field experiments tested three macro-propagation structures (standard chamber, standard chamber with black net, and mulched open bed) and three substrates (loam soil, rice husks, and sawdust). Growth parameters, including leaf number, plant height, root number, root length, and number of suckers harvested, were recorded, while regression, correlation, and principal component analyses were conducted. The standard chamber produced the tallest plants (20.50 ± 0.83 cm) and highest sucker number (102.22 ± 14.09), whereas sawdust and loam soil supported the highest sucker yields (85.33 ± 13.81 and 79.00 ± 11.95, respectively). Rice husks promoted the longest roots (11.49 ± 0.69 cm). The combination of standard chamber with sawdust maximized plant height (22.33 cm) and sucker yield (135.33). Regression analysis revealed a strong positive relationship between decapitated primary suckers and secondary sucker production (R² = 0.741, p < 0.001), confirming that decapitation enhances sucker proliferation. Principal component analysis showed that shoot and sucker traits were the main contributors to variation among treatments, while root traits varied inversely with aboveground growth. These results demonstrate that optimizing macro-propagation structures and substrates, together with sucker decapitation, can significantly improve banana sucker production, thereby increasing the availability of quality planting materials in Malawi.

## 1 Introduction

Bananas (*Musa spp*.) are a crucial crop in global agriculture, playing a significant role in food security and economic stability, especially in tropical and subtropical regions [1]. Banana is one of the major staple and cash crops in Malawi, with an estimated annual production of approximately 695,000 metric tons from about 59,000 hectares [2]. Despite its importance, the production of banana in Malawi is declining due to diseases, poor agronomic practices and lack of disease-free planting materials. Diseases such as banana leaf streak (Black sigatoka) and banana bunchy top disease (BBTD), as well as pests like the corm weevil (Cosmopolites sordidus), nematodes, and banana aphid (Pentalonia nigronervosa), are easily transmitted from one farmer to another. This is largely because the crop is propagated vegetatively, with farmers sharing suckers [3]. Currently almost 70% of bananas in Malawi have been wiped out due to diseases and pests, mainly transmitted through sharing or purchase of infected planting materials. The situation has become so dire that over 70% of the desert bananas consumed in the country are imported from Tanzania [3]. Agricultural extension advice for farmers to uproot and burn infected plants has mostly been ignored due to labour implications involved in destroying banana marts and limited access to clean planting materials to replace the ones uprooted. The lack of clean planting materials has also negatively affected adherence to phytosanitary measures by Department of Agricultural Research Services (DARS) of Malawi.

Tissue culture remains a popular method for rapidly producing disease-free banana planting materials. Plants produced from tissue culture are uniform, true-to-type, disease-free, and can be produced regardless of season [4]. However, tissue culture plants are produced in laboratory conditions under sterile media, requiring expertise and expensive equipment. Additionally, plants from tissue culture are prone to somaclonal variation and require a prolonged period of hardening before they can be accessed by farmers in the field [5]. Therefore, for the case of Malawi where banana production is largely done by resource-constrained smallholder farmers, there is need to explore affordable and sustainable methods of producing disease-free banana planting materials [1].

Macropropagation has emerged as a method that can effectively compliment tissue culture efforts in Malawi. In macropropagation, healthy corms are dug out, prepared to the appropriate size, sterilized, and covered with plastic sheets after being placed in locally available media [6]. These corms produce plantlets within six weeks. Although the number of plants produced is not as high as that produced in tissue culture, the plants produced in macropropagation are simple and take a shorter period to harden. In addition, the plants from macropropagation, just like those from tissue culture, are free from pests and diseases [1]. Importantly, macropropagation is particularly applicable to smallholder farmers in Malawi, as it is a simple technique that requires affordable and locally available materials [6]. Therefore, macropropagation has potential to improve sustainable access of planting materials within local communities.

Although macropropagation is more affordable than tissue culture, the standard recommended unit susing wooden planks, thick plastic sheets, and sawdust as a substrate remain cost-prohibitive for many resource-poor farmers [7]. Previous attempts to construct units from local materials have shown limited adoption, primarily due to low availability of sawdust as a standard substrate [7]. In Ethiopia, simpler macropropagation methods for enset produced up to 80% plantlet emergence using soil and farmyard manure with mulch, demonstrating the potential of low-cost, locally adapted approaches. Recent studies on banana propagation have emphasized the use of locally available substrates to achieve 70–85% sucker multiplication, highlighting the effectiveness of low-cost methods [8,9,10]. To improve adoption in smallholder communities such as those in Malawi, alternative propagation methods that are affordable, require minimal technical skill, and utilize accessible materials are urgently needed. This study addresses this gap by systematically evaluating multiple macropropagation structures and substrates, while quantitatively analyzing sucker proliferation dynamics, to identify scalable, cost-effective strategies for banana planting material production under smallholder.

## 2 Materials and methods

### 2.1 Source of plant materials

The Banana corms (*cv. Williams*) of sword suckers used in this study were sourced from Lilongwe University of Agriculture and Natural Resources (LUANAR) Bunda College Horticulture Farm, which was established two years earlier with virus-free tissue-culture sourced suckers. At the time of sourcing the corms, there were no visible signs of banana bunchy top virus (BBTV) or any other diseases in the banana field.

### 2.2 Experimental site

Macropropagation experiments were conducted at Lilongwe University of Agriculture and Natural Resources (LUANAR) (14°11′S, 33°46′E) from 6 June 2022–19 October 2022, spanning a total of 135 days from corm placement in the propagation chambers to the harvest of final suckers. The site is located at an altitude of 1100 meters above sea level within the mid-elevation upland plateau agro-ecological zone of Malawi. The mean annual temperature at the site is 19.1°C, with a recorded maximum of 27.8°C and minimum of 17.2°C.

### 2.3 Experimental design

#### 2.3.1 Experimental layout.

The substrates investigated in the study are rice husks (S1), sawdust (S2), and loam soil (S3). The study included three structural setups: a standard chamber (SC), a standard chamber with a black net (SBN), and mulched open bed (MOB). The decision to include black netting was based on our initial studies, which revealed that plastic without netting was causing a hot microclimate that resulted in scorching of the suckers. The experimental design followed a Split Plot Design with three replicates and eight corms per replicate. Conditions in the SC and SBN, including temperature, and humidity were recorded. The primary aim of the study is to identify optimal conditions for macropropagation using accessible and cost-effective materials. The statistical model employed for the analysis is expressed as follows:


$$\begin{array}{c} {\text{Y}}_{\text{ijk}}=\mu +{\text{A}}_{\text{i}}+{\text{B}}_{\text{j}}+{\left(\text{AB}\right)}_{\text{ij}}+\varepsilon_{\text{ijk}} \end{array}$$ (1)

Where: Y_ijk_ = the response variable for the k^th^ replicate of the i^th^ main plot and j^th^ sub-plot, μ = overall mean, A_i_ = effect of the i^th^ level of the main plot factor (macro-propagation structure), B_j_ = effect of the j^th^ level of the sub-plot factor (substrate), (AB)_ij_ = interaction effect between the main plot and sub-plot factors, ϵ_ijk_ = random error term associated with the k^th^ replicate of the i^th^ main plot and j^th^ sub-plot, assumed to be normally distributed.

#### 2.3.2 Macropropagation structures and management.

**Fig 1:**
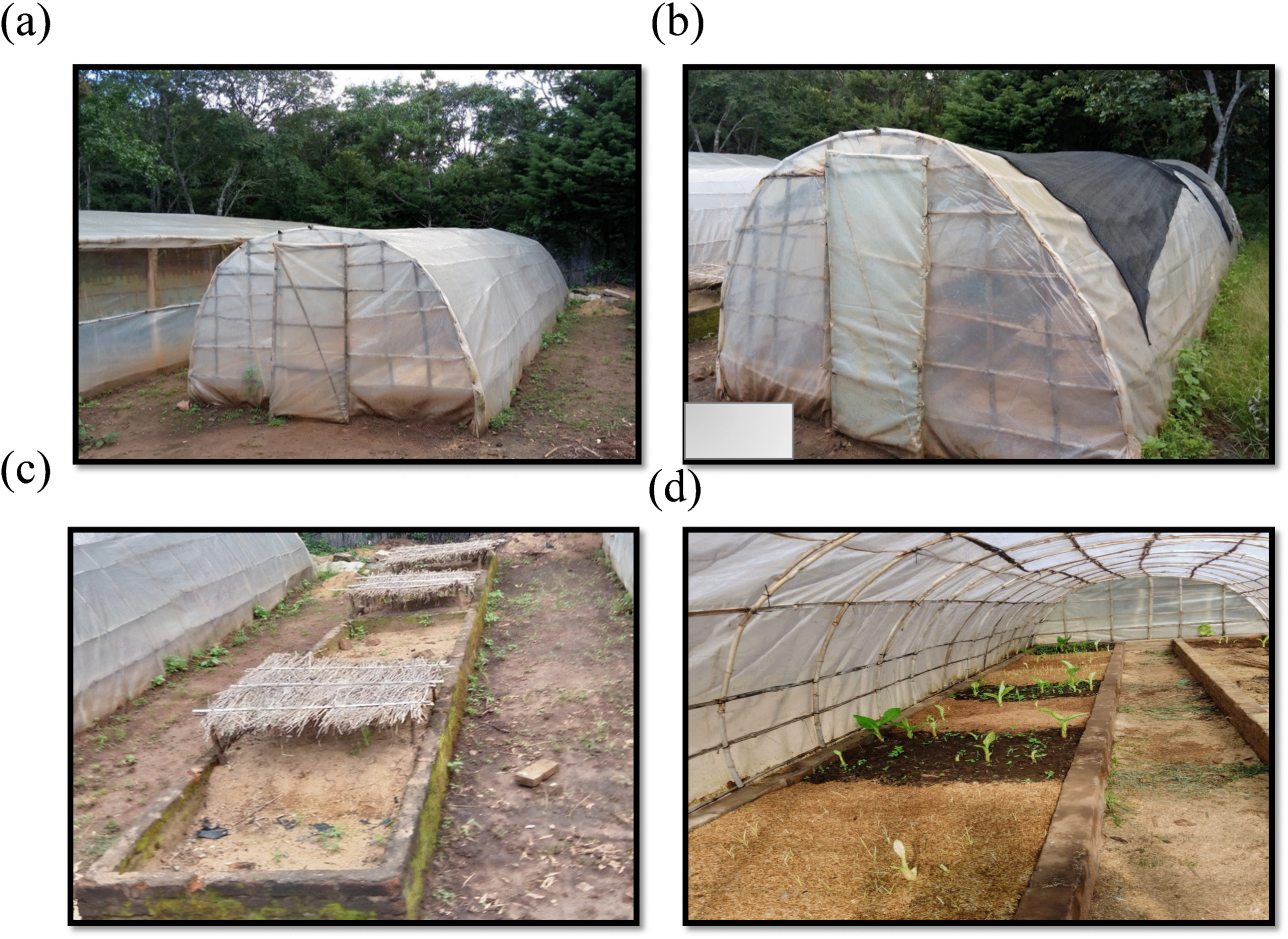
Macropropagation Structures. **(a)** Standard Chamber (SC), **(b)** Standard chamber with a black net (SBN), **(c)** Mulched open bed (MOB), and **(d)** Substrate allocation.

During the banana macropropagation period (July–October 2025), microclimatic conditions varied among the propagation structures, with morning and afternoon temperatures and relative humidity summarized in Table 1. The open tunnel experienced the highest temperature fluctuations, particularly in the afternoons, with temperatures frequently exceeding 29°C and lower relative humidity, whereas the humidity chamber and humidity chamber with black net maintained more stable temperature regimes and consistently higher relative humidity, especially during morning hours, likely supporting improved sucker growth. The propagation structures consisted of three identical beds measuring 8 m long, 1 m wide, and 30 cm high, built with bricks, sand, and cement, with quarry stones to separate soil and substrate and enhance drainage. Two standard structures (standard chamber and Standard Chamber + Net) were constructed using bamboo sticks covered with clear plastic sheets (Fig 1a and 1b), each 1.8 m high, 2.5 m wide, and 8 m long, with one covered in black net to form the humidity chamber with black net (Fig 1b). A third smaller structure, 0.5 m high, was made from poles, nails, and strings and thatched with grass (Fig 1c). Each 8 m bed inside and outside the humidity chambers was divided into nine 1 m² beds, with the standard chamber (SC) serving as the control. Temperature and humidity meters were placed in all structures to monitor conditions, and raw daily weather data are provided in S1 Table.

**Table 1:** Average temperature and humidity conditions inside different macro-propagation structures over the study period.

Structure	Morning Temp (°C)	Afternoon Temp (°C)	Morning Humidity (%)	Afternoon Humidity (%)
Standard Chamber	21.2 ± 2.0	21.9 ± 2.8	42.8 ± 10.7	43.0 ± 12.3
Standard Chamber + Net	21.9 ± 1.6	27.9 ± 7.8	75.0 ± 9.0	78.8 ± 5.2
Open Tunnel	30.8 ± 4.0	29.2 ± 5.5	81.4 ± 8.4	73.8 ± 6.1
Outside	21.2 ± 1.7	23.0 ± 2.9	46.4 ± 12.4	43.2 ± 17.2

#### 2.3.3 Substrate preparation and characterization.

Three types of substrate were used in this study: sawdust, rice husks, and loam soil (S1 Fig). Fresh sawdust was obtained from local timber processors, while fresh rice husks were collected from rice mills in the same area. Loam soil was sourced from Bunda College forest. The samples of the substrates were sent to the soil lab for water holding capacity characterization. The air-dried substrates were packed gently in core rings (5.3 diameter and 6 length) in 3 replicates. The rings were placed on a pressure plate and water was applied gently around the rings. The rings were left for 48 hours until saturation (water appeared at the surface). The rings were then immediately placed on ceramic plates in a pressure plate apparatus (pressure plate extractor) and subjected to pressures of 33 kPa (field capacity) and 1500 kPa (permanent wilting point) to determine substrate water retention. Water content at each suction was determined gravimetrically (Table 2). Each substrate was sterilised by placing it in a sack and hanging in a drum of 200-litres capacity. The sack did not touch the bottom of the the drum. The drum was then filled with about 30 litres of water and closed. These were heated for about six hours so that the heat from the boiling water could kill bacteria, fungus, nematodes, weevils and other insects within the substrate. The sterilised substrates were then cooled. Each cooled substrate was used to fill a 8m^2^ beds, as illustrated in Fig 1d.

**Table 2:** Physical characterization of substrate water holding capacity at field capacity (ϴ_*fc*_) and permanent wilting point (ϴ*_pwp_*).

Substrate	ϴ_*fc*_ (%)	ϴ*_pwp_* (%)
Rice husk	71.5	58.9
Saw dust	109.9	92.1
Loam soil	25.6	9.9

Values are presented as descriptive measurements of substrate physical properties and were not subjected to statistical analysis.

#### 2.3.4 Banana corms preparation and planting.

**Fig 2:**
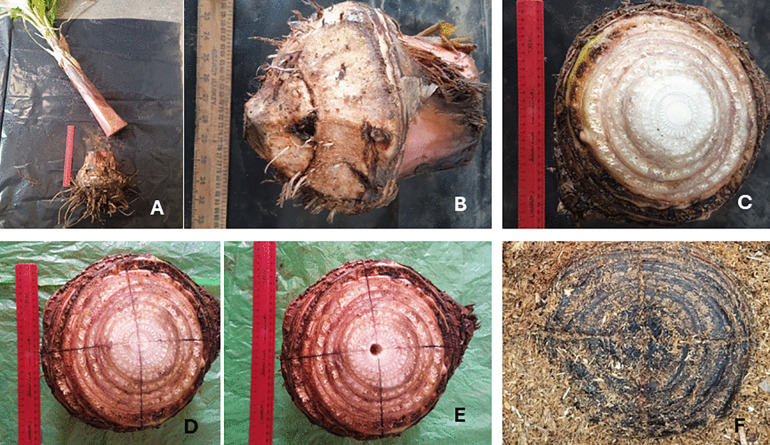
Preparation of corm for macropropagation. **A:** Corm with pseudostem removed; **B:** Corms were prepared using a panga knife, a large, broad-bladed cutting tool similar to a machete, commonly used in East and Southern Africa for agricultural purpose to remove weevils, weevil eggs, and nematodes; **C:** Corm with peeled leaf sheaths guided by the V-junction of leaves to expose lateral buds; **D:** Corm with central meristem scarified by making two deep cross-wise incisions to destroy apical dominance; **E:** Central meristem destroyed by mechanical removal through screwing with sharp knife on the centre of the pseudo stem to remove any apical dominance; **F:** Corm placed 3 cm deep in sawdust in humid chamber.

Banana Cv. Williams Corms were obtained from sword suckers. Corms were obtained from farms that did not have visibly detectable pests and diseases. The farms had been established from tissue-culture sourced suckers. The banana corms were processed using the whole corm method, where roots and excess pseudo stem were removed, and apical dominance was eliminated [11] (Fig 2B and 2C). A sharp knife was was used to make incisions on top of the corm. These incisions were 5 cm deep into the corm. The incisions were made in such a way that the two lines crossed at the center of the corm, forming the pattern of a cross. The central meristem was killed by inserting a knife vertically and screeing into the flesh of the corm until the knife had transitioned from the pseoudo-leaf section and reached the underground stem section (Fig 2D and 2E). The circumference, height, and diameter of all corms were similar. Subsequently, the corms were sterilized by immersing them for 15 minutes in a solution prepared by dissolving 100 g of 50% Copper Oxychloride powder in 10 L of water. After sterilization, the corms were cured under a shed for 48 hours before being transferred to three propagation structures filled with sawdust, rice husks, and loam soil. Corms were spaced 5 cm apart, and covered with a 2–5 cm layer of the respective medium (Fig 2F). Eight corms prepared as above were planted in each replicate of 8 m^2^ bed. The substrates were watered to field capacity throughout the macropropagation period.

#### 2.3.5 Decapitation process.

Removal of the corm’s central meristem stimulated some lateral buds to rapidly produce primary suckers within four weeks. Primary suckers that had a large base at the connection to the corm were decapitated when they had two or three leaves. Decapitation involved cutting the pseudostem of the sucker at 2 cm distance from the connection to the corm. This was followed by scarifying the central meristem of the primary sucker by making two deep cross-wise incisions to destroy apical dominance (Fig 2E). Primary suckers that had a narrow connection to the corm were harvested even when only one root was present. This was done to induce other dormant lateral buds from producing shoots before the corm lost vigour in propagation.

### 2.4 Data collection and analysis

Growth and yield parameters were recorded for each corm and its suckers, including the number of leaves, plant height, root number and length for primary suckers, and leaf number, height, and girth for decapitated suckers. In addition, the numbers of shoot sprouts, decapitated suckers, primary and secondary suckers harvested, and the total harvested suckers were recorded. The collected data were analyzed using ANOVA for a split-plot design in Genstat 18th Edition [12], with macropropagation structures assigned to main plots and substrates to subplots. Tukey’s test at a significance level of 0.05 was used to separate means.

Multivariate analyses were conducted using R software [13]. Principal component analysis (PCA) was employed to explore relationships among measured traits and to identify major sources of variation within the dataset. The correlation matrix of selected quantitative variables was first computed using the cor() function. The suitability of the dataset for PCA was evaluated using the Kaiser–Meyer–Olkin (KMO) measure of sampling adequacy and Bartlett’s test of sphericity, implemented through the KMO() and cortest.bartlett() functions from the psych package. Only datasets meeting the assumptions of adequate sampling and significant sphericity were retained for PCA.

PCA was performed using the PCA() function from the FactoMineR package with data standardized (*scale.unit* = TRUE) to account for differences in measurement scales. Eigenvalues and the proportion of variance explained by each principal component were extracted, and components with eigenvalues greater than 1 were retained according to Kaiser’s criterion. Scree plots were generated using the fviz_eig() function from the *factoextra* package [14] to visualize the contribution of each component to total variance. Factor loadings and individual scores were obtained to assess the contribution of each trait and to examine multivariate patterns in sucker growth and yield-related traits.

Pearson’s correlation analysis was conducted using the *corrplot* [15] and *metan* packages [16] to quantify linear associations among variables and to visualize correlation structures. In addition, simple linear regression analyses were performed using the *stats* package in R [13] to model relationships between selected traits and to further elucidate their effects on growth performance.

## 3 Results

### 3.1 Effect of macropropagation structure

The study compared the effect of different macropropagation structures on growth parameters of primary suckers, specifically analyzing the standard chamber (SC), standard chamber with a black net (SBN), and mulched open bed (MOB) (Table 3). The number of leaves per plant did not vary significantly among the structures (False Positive Rate (Fpr.) = 0.159), with the MOB having the highest mean (4.76 ± 0.15) and the Standard Chamber the lowest (4.03 ± 0.14). Plant height exhibited significant variation (Fpr. = 0.011), with the standard chamber and standard chamber with a black net structures producing taller plants (20.50 ± 0.83 cm and 20.26 ± 1.02 cm, respectively) compared to the mulched open bed (16.59 ± 0.79 cm). The number of roots also showed significant differences (F probability (Fpr.) = 0.010), with the standard chamber having the highest (6.78 ± 0.52) and the standard chamber with a black net the lowest (4.18 ± 0.52). Root length did not vary significantly (Fpr. = 0.545), ranging from 7.78 ± 1.04 cm in standard chamber with a black net to 9.25 ± 1.09 cm in the mulched open bed. The number of harvested suckers exhibited a highly significant difference (Fpr. < 0.001), with the standard chamber yielding the most (102.22 ± 14.09) compared to standard chamber with a black net (59.11 ± 7.32) and the mulched open bed (38.33 ± 3.78). Overall, the grand mean values were 4.42 ± 0.26 leaves, 19.12 ± 0.92 cm height, 5.35 ± 0.56 roots, 8.73 ± 1.04 cm root length, and 66.60 ± 9.84 harvested suckers, with the highest coefficient of variation observed for the number of harvested suckers (44.3%) and the lowest for the number of leaves (17.6%). The least significant difference values highlighted the statistical significance thresholds for each measured trait (Table 3).

**Table 3:** Effect of Macropropagation Structures on primary suckers.

Macropropagation Structure	No. of leaves	Plant height (cm)	No. of roots	Root Length (cm)	No. of Harvested suckers
Standard Chamber	4.03 ± 0.14^a^	20.50 ± 0.83^b^	6.78 ± 0.52^b^	9.15 ± 0.85^a^	102.22 ± 14.09^b^
SBN	4.48 ± 0.44^a^	20.26 ± 1.02^b^	4.18 ± 0.52^a^	7.78 ± 1.04^a^	59.11 ± 7.32^a^
Open Bed	4.76 ± 0.15^a^	16.59 ± 0.79^a^	5.08 ± 0.60^b^	9.25 ± 1.09^a^	38.33 ± 3.78^a^
Grand Mean	4.42 ± 0.26	19.12 ± 0.92	5.35 ± 0.56	8.73 ± 1.04	66.60 ± 9.84
HSD	0.762	2.708	1.631	3.041	28.85
CV%	17.6	14.5	31.2	35.6	44.3
Fpr.	0.159	0.011	0.010	0.545	<.001

Superscript letters: Indicate statistically significant differences among treatments as determined by Tukey's test at a significance level of 0.05. Values followed by the same letter within a column are not significantly different from each other.

### 3.2 Effect of substrate

The effect of substrate on various growth parameters of primary suckers were assessed using loam soil, rice husks, and sawdust (Table 4). Significant differences were observed in plant height (Fpr = 0.013) and root length (Fpr < 0.001) among the substrates. Suckers grown in loam soil and sawdust exhibited similar plant heights (20.44 ± 0.64 cm and 20.27 ± 1.04 cm, respectively), which were significantly greater than those grown in rice husks (16.27 ± 0.96 cm). Root length was longest in suckers grown in rice husks (11.49 ± 0.69 cm), significantly surpassing those in loam soil (6.28 ± 0.38 cm) and sawdust (8.40 ± 0.91 cm). The number of leaves and roots per sucker did not differ significantly among substrates (Fpr = 0.690 and Fpr = 0.284, respectively). However, the number of harvested suckers varied significantly (Fpr = 0.009), with sawdust (85.33 ± 13.81) and loam soil (79.00 ± 11.95) producing more suckers compared to rice husks (35.33 ± 3.62). These findings underscore the differential effects of substrate on growth parameters of primary suckers, highlighting implications for optimizing cultivation practices in similar agricultural contexts.

**Table 4:** Effect of substrate on total banana suckers.

Substrate	No. of leaves	Plant height (cm)	No. of roots	Root Length (cm)	No. of Harvested suckers
Loam Soil	4.23 ± 0.21^a^	20.44 ± 0.64^b^	5.44 ± 0.54^a^	6.28 ± 0.38^a^	79.00 ± 11.95^b^
Rice Husks	4.55 ± 0.24^a^	16.27 ± 0.96^a^	6.04 ± 0.85^a^	11.49 ± 0.69^b^	35.33 ± 3.62^a^
Saw Dust	4.49 ± 0.34^a^	20.27 ± 1.04^b^	4.56 ± 0.43^a^	8.40 ± 0.91^a^	85.33 ± 13.81^b^
Grand Mean	4.42 ± 0.28	19.12 ± 0.932	5.35 ± 0.65	8.73 ± 0.72	66.60 ± 11.22
HSD	0.815	2.732	1.895	2.099	32.92
CV%	18.8	14.6	36.3	24.6	50.6
Fpr.	0.690	0.013	0.284	<.001	0.009

Superscript letters: Indicate statistically significant differences among treatments as determined by Tukey's test at a significance level of 0.05. Values followed by the same letter within a column are not significantly different from each other.

### 3.3 Combined effects of Macropropagation structure and substrate

Two-way analysis revealed significant main and interactive effects of macropropagation structure and substrate on key growth and productivity parameters of primary suckers (Table 5). Macropropagation structure exerted a significant main effect on plant height, root traits, and number of harvested suckers (Fpr. ≤ 0.001), with standard chamber systems consistently outperforming open-bed systems. Substrate type also showed a significant main effect on crop performance, indicating differential responses among loam soil, rice husks, and sawdust across measured traits. Importantly, a significant structure × substrate interaction was observed for plant height, number of roots, root length, and number of harvested suckers (Fpr. < 0.001), demonstrating that substrate effects were dependent on the macropropagation structure employed. The tallest plants and highest sucker yield were recorded in the standard chamber combined with sawdust (22.33 cm and 135.33 suckers), whereas the lowest performance was observed in open-bed and black-net systems combined with rice husks.

**Table 5:** Combined effects of macropropagation structure and substrate on growth and yield parameters of primary and decapitated suckers.

Macropropagation structure	Substrate	No. of leaves	Plant Height (cm)	No. of roots	Root length (cm)	No. of harvested Suckers
Standard chamber	Loam Soil	3.72^a^	21.03^b^	6.35^ab^	6.84^abc^	124.33^d^
	Rice husks	4.00^a^	18.13^ab^	8.43^b^	11.83^c^	47^ab^
	Saw Dust	4.38^a^	22.33^b^	5.56^ab^	8.78^abc^	135.33^d^
Standard Chamber with Black Net	Loam Soil	4.24^a^	20.71^b^	5.08^ab^	6.17^ab^	66.33^bc^
	Rice husks	5.07^a^	18.36^ab^	3.38^a^	11.49^bc^	32^a^
	Saw Dust	4.97^a^	21.70^b^	4.07^a^	5.70^a^	79^c^
Open Bed	Loam Soil	4.73^a^	19.58^b^	4.88^ab^	5.85^ab^	46.33^ab^
	Rice husks	4.59^a^	13.40^a^	6.31^ab^	11.16^abc^	27^a^
	Saw Dust	4.12^a^	16.78^ab^	4.04^a^	10.73^abc^	41.67^a^
	Mean	4.42 ± 0.48	19.12 ± 1.17	5.36 ± 0.86	8.73 ± 1.12	66.6 ± 4.79
	HSD	1.429	3.512	2.583	3.367	14.25
	CV%	18.7	10.6	27.9	22.3	12.5
	Fpr.	0.594	0.001	<.001	<.001	<.001

Superscript letters: Indicate statistically significant differences among treatments as determined by Tukey's test at a significance level of 0.05. Values followed by the same letter within a column are not significantly different from each other.

### 3.4 Regression analysis of the relationship between decapitated suckers and secondary suckers

**Fig 3:**
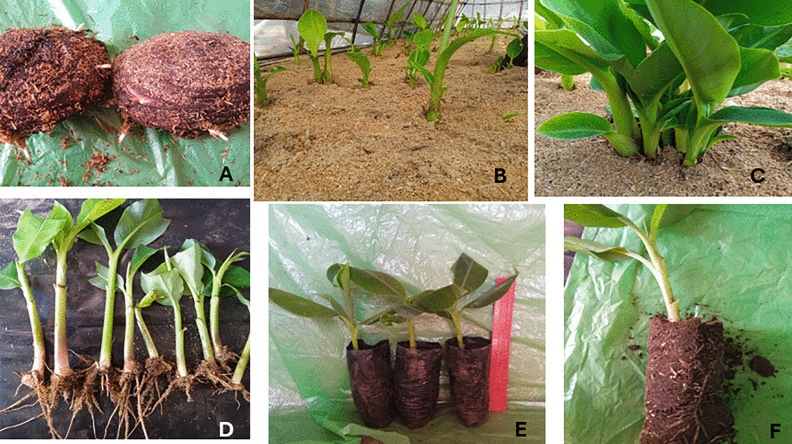
Banana development process in sawdust after decapitation of primary suckers. **A:** Corms develop roots two weeks after placement in sawdust substrate; **B:** Primary shoots emerge above substrate from two to five weeks; **C:** Decapitated primary shoots develop multiple secondary shoots; **D:** Shoot developing after decapitation are harvested and potted for aclimatisation after developing roots; **E:** Well-acclimatised suckers with three to five leaves after three weeks in pots; **F:** Well-developed roots of banana sucker after successful acclimatisation.

A regression analysis was conducted to investigate the correlation between the number of decapitated suckers and the production of secondary suckers. The independent variable was the number of decapitated suckers, and the dependent variable was the number of secondary suckers. The results, as shown in S2 Table, indicated a significant positive relationship (p < 0.001) with a regression coefficient of 2.867. This suggests that for each additional decapitated sucker, the number of secondary suckers increased by approximately 2.87. The intercept was −0.844, which was not statistically significant (p = 0.843). The R-squared value of 0.741 indicates that 74.1% of the variability in secondary sucker production can be explained by the number of decapitated suckers. The F-statistic of 80.94 (p < 0.001) further supports the significance of the relationship, highlighting the effectiveness of decapitation in promoting sucker proliferation (Fig 3).

### 3.5 Correlation analysis

**Fig 4:**
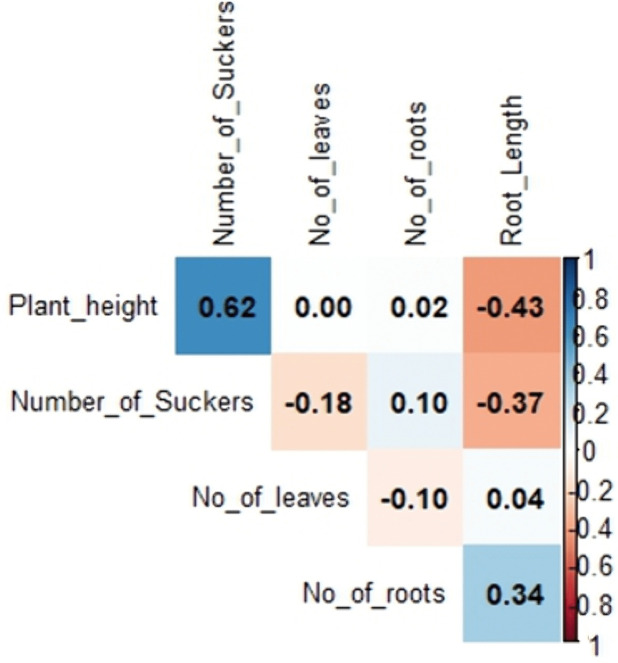
Shows Pearson's correlation coefficients for the variables studied.

Pearson correlation analysis was conducted to assess the relationships among growth parameters of primary suckers, including plant height, number of leaves, number of roots, root length, and total number of suckers (Fig 4). Plant height was positively correlated with the total number of suckers (r = 0.62, p < 0.05) and the number of roots (r = 0.34, p < 0.05), and negatively correlated with root length (r = −0.43, p < 0.05). Root length was also negatively correlated with the total number of suckers (r = −0.37, p < 0.05). The remaining correlations among growth parameters, including the number of leaves, were non-significant (ranging from −0.10 to 0.04).

### 3.6 Principal component analysis

#### 3.6.1 Suitability of the Data for PCA.

##### 3.6.1.1 Kaiser–Meyer–Olkin (KMO) Measure.

The suitability of the dataset for principal component analysis (PCA) was assessed using the Kaiser–Meyer–Olkin (KMO) measure and Measures of Sampling Adequacy (MSA). The overall KMO value was 0.56, indicating marginal sampling adequacy for PCA. Variable-specific MSA values varied among traits, with Root Length (0.61) showing the highest adequacy, while Number of Leaves (0.36) and Number of Roots (0.40) exhibited lower adequacy, suggesting weaker contributions to the correlation structure used in PCA (Table 6).

**Table 6:** Kaiser–Meyer–Olkin (KMO) test and Measures of Sampling Adequacy (MSA) for PCA.

Trait	MSA
No. of leaves	0.36
Plant height	0.59
No. of roots	0.40
Root Length	0.61
Number of Suckers	0.58
**Overall**	0.56

Values represent overall KMO (dataset-level adequacy) and MSA (variable-level adequacy for PCA structure).

##### 3.6.1.2 Bartlett’s test of sphericity.

Bartlett’s test of sphericity was performed to evaluate whether the correlation matrix of the growth and yield traits significantly differed from an identity matrix. The test was significant (χ² = 22.793, df = 10, p = 0.01154), indicating that the variables were sufficiently correlated to justify principal component analysis (PCA). This result confirms that the dataset meets the assumptions required for PCA (Table 7).

**Table 7:** Bartlett’s test of sphericity for growth and yield traits.

Test Statistic	*χ²*	df	p-value
Bartlett’s test	22.793	10	0.01154

#### 3.6.2 Eigenvalues and percentage of variance.

**Fig 5:**
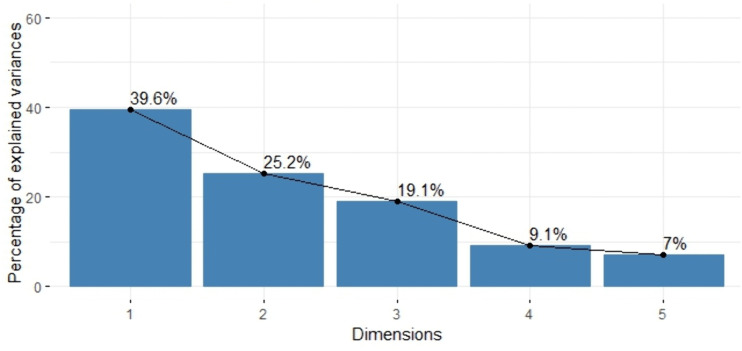
Scree plot showing the proportion of total variance explained by each principal component.

Principal component analysis (PCA) was conducted on the selected growth and yield traits to reduce dimensionality and identify the major sources of variation. The eigenvalues of all principal components were calculated (Table 8). According to Kaiser’s criterion, only the first two components had eigenvalues greater than 1 (PC1 = 1.970, PC2 = 1.261). Scree plot further supported this observation (Fig 5).

**Table 8:** Eigenvalues and percentage of variance explained by principal components obtained from principal component analysis of growth and yield trait.

Principal Component	Eigenvalue	% Variance	Cumulative %
PC1	1.970	39.597	39.597
PC2	1.261	25.230	64.826
PC3	0.954	19.072	83.899
PC4	0.456	9.120	93.018
PC5	0.350	6.982	100

#### 3.6.3 Trait loadings.

The contributions of growth and yield traits to the first four principal components are shown in Table 9, with only loadings ≥ |0.3| presented. PC1 was primarily associated with shoot and sucker production, with strong positive loadings for Plant height (0.843) and Number of Suckers (0.825) and a negative contribution from Root Length (−0.735), indicating that plants with greater shoot and sucker development tended to have shorter roots along this axis. PC2 reflected variation in leaf number and root traits, with negative loading for No. of leaves (−0.477) and positive contributions from No. of roots (0.860) and Root Length (0.425), suggesting a trade-off between leaf and root development. PC3 captured variation in leaf and root proliferation, with positive loadings for No. of leaves (0.856) and No. of roots (0.328), while PC4 was moderately influenced by Root Length (0.500) and negatively by No. of roots (−0.364), representing subtler variation in root architecture. Overall, these four principal components effectively summarized the variation in sucker growth and yield traits, with PC1 and PC2 representing the major axes of variation and PC3 and PC4 capturing more specific patterns in leaf and root development, providing a framework to interpret the effects of structure × substrate treatments.

**Table 9:** Loadings of traits on the first four principal components (PCA). Only loadings ≥ |0.3| are shown.

Trait	PC1	PC2	PC3	PC4
No. of leaves		−0.477	**0.856**	
Plant height	**0.843**			
No. of roots		**0.860**	0.328	−0.364
Root Length	**−0.735**	0.425		**0.500**
Number of Suckers	**0.825**	0.320		

#### 3.6.4 PCA biplot.

**Fig 6:**
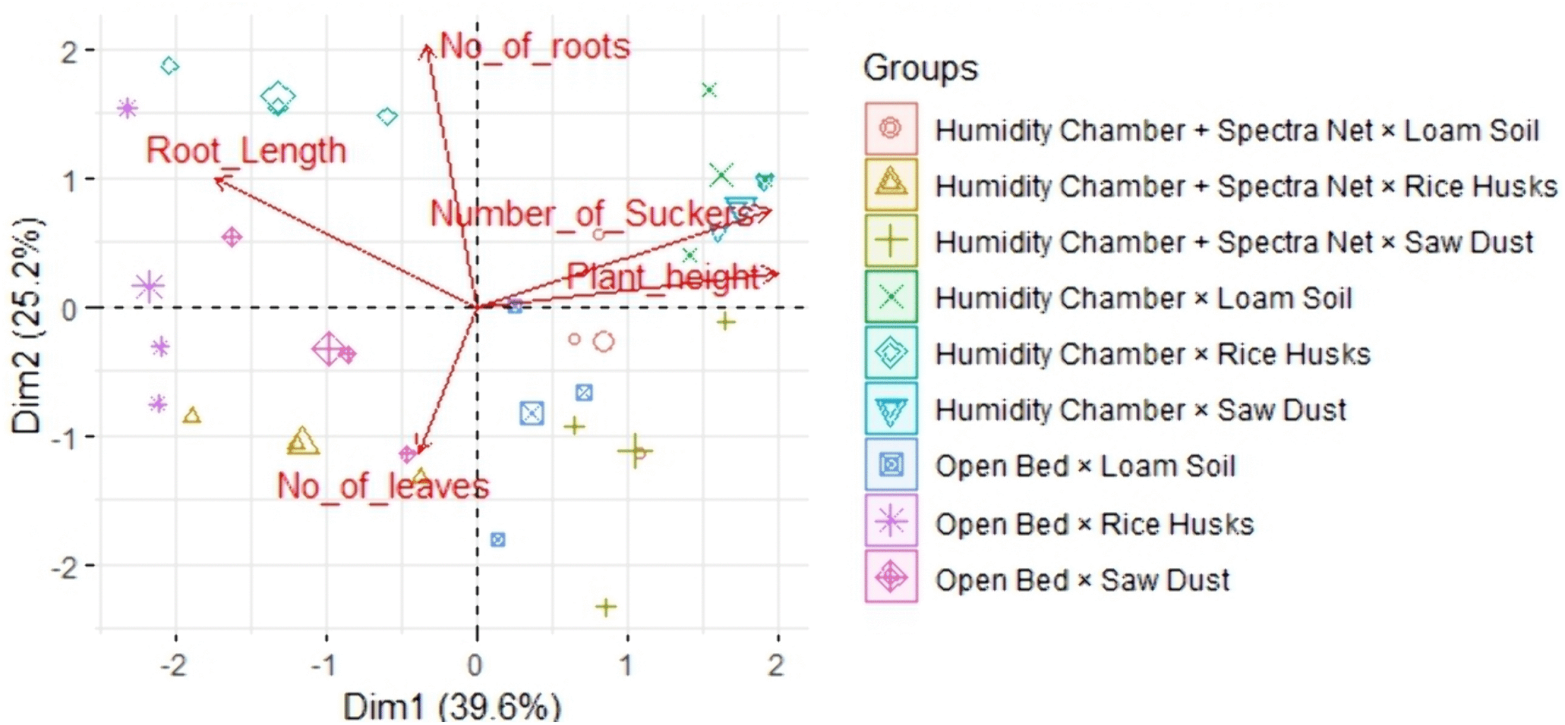
Principal component analysis (PCA) biplot showing relationships among plant growth traits and clustering of treatments defined by combined macro-propagation structures and substrate type.

To examine patterns of association among plant growth traits and to evaluate variation arising from combined macro-propagation structures and substrate types, principal component analysis (PCA) was performed (Fig 6). The first two principal components explained 64.8% of the total variation, with Dim1 and Dim2 accounting for 39.6% and 25.2%, respectively. Dim1 was primarily associated with aboveground growth traits, particularly plant height and number of suckers, while Dim2 was influenced by root-related traits and number of leaves. Combined treatments involving the humidity chamber, especially with loam soil and rice husks, clustered on the positive side of Dim1, whereas open bed–based combinations were more dispersed and showed stronger associations with root length and leaf number. Overall, the PCA biplot indicates that variation in plant growth traits was structured by the combined effects of macro-propagation structures and substrate types.

### 3.7 Economic analysis of banana macropropagation systems

An economic analysis was conducted to assess the economic feasibility of banana macropropagation using different structures and substrates on a 1 m² production-area basis (Table 10). Net returns varied from USD 29.24 to USD 185.03, with benefit-cost (B:C) ratios ranging from 1.87 to 12.73 after prorating chamber construction and operational costs from a total footprint of 24 m². Among controlled environments, the standard chamber with sawdust yielded the highest net return (USD 185.03 m ⁻ ²; B:C = 11.59), followed by the standard chamber with loam soil (USD 171.39 m ⁻ ²; B:C = 12.73), correlating with the highest sucker yields (135 and 124 suckers m ⁻ ², respectively). The use of a black net consistently decreased profitability, resulting in lower B:C ratios (2.56–6.06), with the lowest performance observed for rice husks under the black-net chamber (USD 29.24 m ⁻ ²; B:C = 2.56). Open-bed macropropagation had mixed outcomes; although loam soil had a high B:C ratio (9.58) due to minimal production costs (USD 7.20 m ⁻ ²), the absolute net returns were lower compared to standard chambers.

**Table 10:** Economic analysis of banana macropropagation under different structures and substrates (1 m² basis).

Macropropagation Structure	Substrate	Suckers Harvested	Adjusted Total Cost (USD)	Gross Return (USD)	Net Return (USD)	B:C Ratio
Standard Chamber	Loam Soil	124	14.61	186.00	171.39	12.73
Standard Chamber	Rice Husks	47	16.67	70.50	53.83	4.23
Standard Chamber	Saw Dust	135	17.47	202.50	185.03	11.59
Standard Chamber + Black Net	Loam Soil	66	16.69	99.00	82.31	5.93
Standard Chamber + Black Net	Rice Husks	32	18.76	48.00	29.24	2.56
Standard Chamber + Black Net	Saw Dust	79	19.55	118.50	98.95	6.06
Open Bed	Loam Soil	46	7.20	69.00	61.80	9.58
Open Bed	Rice Husks	27	20.92	40.50	19.58	1.94
Open Bed	Saw Dust	41	32.90	61.50	28.60	1.87

**Note:** Chamber construction and operational costs were incurred for a total chamber area of 24 m² (8 m × 3 m), while production and data collection were carried out on 1 m² experimental units. Consequently, the total costs for chamber-based treatments were adjusted to a 1 m² basis before calculating the net return and benefit-cost (B:C) ratio. Net return is calculated as the Gross return minus the Adjusted total cost, and the B:C ratio is determined by dividing the Gross return by the Adjusted total cost.

## 4 Discussion

Micropropagation through tissue culture techniques is an efficient method of producing large quantities and good quality banana suckers, but is constrained by high capital and skills requirements. As such macropropagation is being advocated as an effective alternative method which requires less capital and skills to produce large numbers of better-quality banana suckers [1]. However, macropropagation is a relatively new technology in Malawi and requires adaptation to ensure that it produces sufficient suckers at a cost and scale accessible to smallholder farmers. In this study, we systematically evaluated multiple macropropagation structures and locally available substrates and found that the standard chamber combined with saw dust produced the highest sucker yield (135 suckers m ⁻ ²) and the greatest net economic return (USD 185.03 m ⁻ ²; B:C = 11.59). Although the standard chamber with loam soil achieved the highest benefit–cost ratio overall (12.73), reflecting superior economic efficiency per dollar invested, its absolute sucker output and net return were slightly lower than those obtained with saw dust. This integrated biological economic assessment provides the first evidence under Malawian conditions that specific combinations of low-cost structures and locally available substrates can substantially enhance both the productivity and profitability of banana planting material production, thereby addressing a key bottleneck in local banana cultivation systems.

This study represents a pioneering evaluation of structured macropropagation systems and substrates aimed at optimizing the growth of Cv. Williams banana suckers, with potential application to other banana cultivars, plantain, and ensete. By systematically comparing various macropropagation structures and substrates, our findings reveal significant differences in growth metrics, underscoring the critical role of macropropagation structures in banana sucker production. Notably, the standard chamber and the standard chamber with black net consistently outperformed the mulched open bed structure, highlighting the efficacy of enclosed and semi-enclosed environments in promoting superior growth outcomes. The superior performance of suckers produced in these structures can be attributed, at least in part, to more favorable and stable microclimatic conditions, particularly higher temperature and relative humidity compared with the open bed system. Enclosed macropropagation structures reduce evaporative losses, buffer temperature fluctuations, and maintain elevated humidity levels, which are essential for bud break, cell expansion, and rapid sucker emergence in bananas, thereby enhancing metabolic activity and meristematic growth [17]. In contrast, the mulched open bed structure is more exposed to ambient environmental fluctuations, resulting in lower and less stable humidity and temperature regimes that can slow bud activation, increase moisture stress, and reduce the efficiency of nutrient uptake, ultimately limiting sucker proliferation and growth performance. These findings align with previous studies emphasizing the importance of warm and humid conditions for optimal banana macropropagation and early vegetative development [18,19].

A novel aspect of our study is the investigation into decapitation as a method to enhance sucker proliferation, providing new insights into banana sucker production practices. We observed a significant positive correlation between decapitated suckers and the subsequent production of secondary suckers, indicating the potential of targeted decapitation to increase sucker yields per banana corm. This innovative approach offers practical strategies to enhance productivity and sustainability in commercial banana farming, advancing our understanding beyond traditional propagation methods. The robustness of this finding is supported by a high R-squared value (0.741) and a significant regression coefficient (2.867), affirming decapitation as an effective technique for enhancing sucker proliferation.

Moreover, our study emphasizes the critical role of substrate selection in banana cultivation, with sawdust identified as highly effective for enhancing sucker yield. The robust support for increased plant height and sucker production in loam soil stems from its favorable physical properties, including optimal porosity and excellent water retention capacity. Furthermore, loam soil exhibits a near-neutral pH of 6.7, a balanced nitrogen content of 0.095%, and a notably high phosphorus content of 113.93 mg/kg (S2 Table). These factors collectively optimize nutrient availability, microbial activity, and root development, crucial for promoting vigorous vegetative growth and sustaining plant health. The balanced texture of loam soil, consisting of sand, silt, and clay, facilitates efficient drainage and adequate water retention, thereby preventing waterlogging and supporting robust growth [20]. This balanced water regime reduces irrigation frequency and minimizes the risk of overwatering, offering practical benefits for sustainable farming practices that enhance productivity while reducing costs [21]. Moreover, the enhanced root environment provided by loam soil contributes to stronger plants capable of withstanding environmental stresses, thereby improving overall plant resilience and health. These findings underscore the importance of considering substrate physical characteristics alongside nutrient content, providing valuable insights for farmers seeking to optimize yield and economic returns through informed substrate selection [1].

In terms of macropropagation structures, our results demonstrate significant variations in plant height, root development, and sucker production. The Standard Chamber consistently produced the tallest plants and highest sucker yields, underscoring its effectiveness due to optimal moisture retention, aeration, and temperature regulation critical for plant vigor. Conversely, the Open Bed with rice husks yielded the shortest plants, suggesting a need for improvements to support overall plant vigor effectively. These findings underscore the necessity of optimizing both environmental conditions and substrate choice to maximize banana cultivation outcomes, providing clear guidance for farmers aiming to enhance yield and economic returns.

The significant macropropagation structure × substrate interaction demonstrates that substrate performance depends strongly on the propagation environment, indicating that neither factor alone is sufficient to optimize growth. The combination of the standard chamber and sawdust produced the tallest plants (22.33 cm) and highest sucker numbers (135.33), reflecting a synergistic effect of a favorable microclimate and optimal rooting medium. Enhanced aeration and moisture retention in sawdust likely complemented the stable temperature and humidity of the standard chamber, promoting root proliferation, nutrient uptake, and subsequent shoot elongation. In contrast, rice husks performed poorly in open-bed and black-net systems, where low water-holding capacity and limited nutrient buffering intensified moisture stress, restricted root growth, and suppressed sucker production. Principal component analysis (PCA) further supports these findings, with treatments involving the standard chamber, particularly with sawdust and loam soil, clustering strongly with aboveground growth traits such as plant height and sucker number, while open-bed combinations were more associated with root length and leaf number. Together, these results underscore the importance of jointly optimizing macropropagation structure and substrate choice, as well as managing key environmental parameters such as temperature and humidity, to maximize banana sucker productivity.

From a systems perspective, these findings indicate that crop productivity is governed by multiple interacting biological and environmental factors, where performance emerges from the combined effects of propagation structure and substrate properties rather than a single independent factor, highlighting the importance of integrated management strategies in macropropagation systems. Optimization techniques are widely applied across scientific disciplines to address complex multi-variable systems involving competing objectives. For instance, multi-objective optimization approaches have been implemented in engineering and energy systems to improve system efficiency, reliability, and performance under multiple constraints, including solar energy systems [22], compressed air energy storage systems [23], and natural gas pipeline networks [24]. These studies demonstrate the effectiveness of optimization frameworks in handling interacting variables and balancing trade-offs in complex systems. In a similar manner, the present study involves interacting biological and environmental factors, where banana sucker productivity is influenced by the combined effects of macropropagation structure and substrate selection, requiring an integrated approach to identify optimal production conditions.

The findings from this study are consistent with and extend upon previous research on the influence of environmental factors on plant growth and macropropagation performance [1]. The strong positive correlations among key growth parameters such as plant height, number of secondary suckers, and number of leaves observed in our study align with the results of Pinar et al. [25], who reported that favorable environmental conditions, particularly optimal temperature and humidity levels, significantly enhance vegetative growth and sucker proliferation in banana plants [26]. Similarly, our observation that higher temperatures positively correlate with vegetative growth metrics echoes the findings of Turner et al. [27], Joshi et al. [28], and Hatfield & Prueger. [29], who found that elevated temperatures within a specific range promote cell expansion and leaf area development in banana and maize, respectively.

The dual nature of humidity, with its positive correlation to some metrics and negative impact on plant height, corroborates the nuanced findings of previous studies [30]. For instance, while high humidity can enhance leaf turgor and overall plant biomass, it can also lead to reduced stem elongation and increased susceptibility to fungal diseases [31,32]. This dual impact underscores the necessity of balanced humidity management to optimize growth without incurring adverse effects.

Overall, the alignment of our findings with prior research underscores the importance of integrated environmental management in macropropagation units. The study confirms that both temperature and humidity play crucial roles, and their combined effects need careful balancing to maximize the growth and productivity of macropropagation efforts. These insights not only validate previous studies but also provide a more comprehensive understanding of the environmental dynamics influencing macropropagation, with practical implications for improving agricultural practices and optimizing plant production.

## 5 Conclusions

In conclusion, the study demonstrates that both the macropropagation structure and substrate significantly influence the growth and number of primary banana suckers. The standard chamber proved to be the most effective structure in promoting sucker production, resulting in the highest number of harvested suckers. The addition of a black net to the standard chamber did not show significant improvements in plant performance. Conversely, the mulched open bed, while encouraging leaf production, led to lower number of banana suckers and reduced plant height. The choice of substrate also played a crucial role in plant growth, with loam soil and sawdust proving beneficial for plant height and sucker production. In contrast, rice husks promoted better root growth but resulted in lower overall sucker production. The combination of the standard chamber with sawdust yielded the best results in terms of plant growth and yield, producing the highest number of harvested suckers. Additionally, the regression analysis revealed a positive correlation between decapitated suckers and secondary sucker production, highlighting the importance of decapitation in enhancing sucker proliferation. These findings provide valuable insights for enhancing banana macropropagation techniques to improve sucker production and overall banana cultivation in similar agricultural settings. Future research should focus on investigating the long-term effects of these treatments and the underlying mechanisms influencing growth patterns.

## Supporting information

S1 Fig Different substrates used in the study. (TIF)

S1 Table Weekly temperature and relative humidity data recorded during banana macropropagation. (XLSX)

S2 Table Regression Analysis of the Relationship between Decapitated Suckers and Secondary Suckers. (DOCX)

S1 Raw Data (XLSX)
