# Supplementary material for: Optimization of banana sucker production using different macropropagation structures and substrates
Source: PLoS One. 2026 Jun 22;21(6):e0352099. doi: 10.1371/journal.pone.0352099 (PMC13286141; doi:10.1371/journal.pone.0352099)
Supplement: S2 Table — (DOCX) [file pone.0352099.s003.docx]

**Table S2.** Regression Analysis of the Relationship between Decapitated Suckers and Secondary Suckers

| Parameter | Estimate | Std. Error | t-Value | P-value | 95% confidence interval |
| --- | --- | --- | --- | --- | --- |
| Intercept | -0.844 | 4.221 | -0.200 | 0.843 | -9.523,7.836 |
| Decapitated suckers | 2.867 | 0.318 | 8.933 | <.001 | 2.213, 3.520 |
| R-squared | 0.741 |  |  |  |  |
| Adj. R-squared | 0.732 |  |  |  |  |
| F-statistic | 80.94 |  |  | <.001 |  |
| Prob(F-Statistic) |  |  |  | <.001 |  |
